# Validation of numerical simulations and experiments on impulse characteristics induced by self-excited oscillation

**DOI:** 10.1038/s41598-024-56187-y

**Published:** 2024-03-06

**Authors:** Qiang Wu, Guodong Ji, Jian Zhao, Liudang Sun, Dapeng Han, Li Liu, Huaigang Hu, Jinping Yu, Changchang Chen, Yuqi Sun, Jinyong Guo

**Affiliations:** 1 CNPC Engineering Technology R&D Company Limited, CNPC, Beijing, 102206 China; 2 Dongying Academy of Science and Technology, China University of Petroleum, Shandong, 257061 China; 3 College of Petroleum Engineering, China University of Petroleum, Shandong, 266580 China; 4 CNPC Technology and Development Corporation, CNPC, Beijing, 100009 China; 5 PetroChina Changqing Oilfield Company, CNPC, Shanxi, 710016 China; 6 International Engineering Company, BHDC, Tianjin, 300450 China

**Keywords:** Self-excited oscillation, Resonance rock-breaking, Impulse characteristics, Large eddy simulation, Parameters optimization, Mechanical engineering, Hydrology

## Abstract

The high-frequency pulse flow, equivalent to the natural frequency of rocks, is generated by a self-excited oscillating cavity to achieve resonance rock-breaking. The flow field and oscillating mechanism of the self-excited oscillating cavity were simulated using the large eddy simulation method of Computational Fluid Dynamics (CFD). A field-scale testing apparatus was developed to investigate the impulse characteristics and verify the simulation results. The results show that the fluid at the outlet at the tool is deflected due to the pulse oscillation of the fluid. The size and shape of low-pressure vortices constantly change, leading to periodic changes in fluid impedance within the oscillating cavity. The impulse frequency reaches its highest point when the length–diameter ratio is 0.67. As the length–diameter ratio increases, the tool pressure loss also increases. Regarding the cavity thickness, the impulse frequency of the oscillating cavity initially decreases, then increases, and finally decreases again. Moreover, both the impulse frequency and pressure loss increase with an increase in displacement. The numerical simulation findings align with the experimental results, thus confirming the validity of the theoretical model. This research provides theoretical guidance for the practical application of resonance rock-breaking technology.

## Introduction

In the oil and gas industry, drilling operations in deep and hard formations are becoming increasingly common. However, enhancing rock-breaking efficiency in these formations poses a significant challenge^1–4^. The target rocks for deep drilling include volcanic rock, granite, conglomerate, etc., which are characterized by high abrasiveness, hardness, and uniaxial compressive strength exceeding 200 MPa. Resulting in fast bit wear and low rock-breaking efficiency are observed^5–7^. Therefore, there is an urgent need to explore new and efficient rock-breaking technologies. One such method is resonance rock-breaking technology, which has been proposed to improve rock-breaking efficiency in deep and hard formations^8,9^. By applying the rock’s periodic vibration, resonance can be achieved when the applied vibration frequency matches the natural frequency of the rock^10^. This phenomenon significantly reduces the rock’s failure strength and improves rock-breaking efficiency^11^. The self-excited oscillating induced by hydraulic power is capable of generating high-frequency pulsating jets. The self-excited oscillator is widely utilized for surface cleaning and blockage removal in producing oil wells due to its simple structure, compact size, and absence of additional excitation sources^12–14^. The self-excited oscillating pulse jet’s high pulse pressure and resonance effect can effectively reduce the coal rock breaking threshold pressure and significantly improve the coal rock breaking efficiency^15^. When the pulse frequency of the waterjet is closer to the natural frequency of the rock mass, the resonance effect generated can intensify the degree of rock failure. When the frequency of the pulsed water jet is within a specific range, the rock-breaking impact of the pulsed water jet is significantly better^16^.

Numerous studies have been conducted to examine the properties of waterjet flow and resonance effect, and their effectiveness in breaking rocks. The impact capacity of the waterjet flow and the relaxation of coal can be quantified by evaluating the degree of disturbance and coal relaxation, respectively^17^. Furthermore, a theoretical model has been developed and experimentally validated to determine the self-propelled force of a multi-orifice nozzle^18^. The use of a supercritical CO_2_ jet has shown a gradual increase in rock-breaking efficiency with higher jet pressure^19^. Additionally, Dehkhoda and Hood^20^, by controlling the number of impacts, have shown that the pulsation frequency directly influences the failure zone on the impact surface and that internal cracks tend to propagate in the direction of the principal stress. When the pressure was below 50 MPa and the traverse speed exceeded 600 mm/min, the water jet resulted in minimal damage to the coating^21^. Raghu Prasad and Vidya Sagar^22^ similarly developed a correlation between fracture energy and acoustic emission energy. Experimental findings have also indicated that increasing the jet pressure results in a decrease in residual stress at the stainless steel surface while increasing the cutting speed elevates the residual stress^23^. Furthermore, both impact depth and rock-breaking volume have been observed to increase with higher water inlet velocity^24^. The incorporation of circular grooves in the inlet and outlet sections has proven effective in reducing fluid resistance^25^. Moreover, the erosion depth has been found to increase proportionally with pump pressure; however, the erosion depth of a convergent-straight nozzle is significantly lower than that of a convergent–divergent nozzle^26^. The center impact pressure of the circular nozzle was found to be highest when the inlet pressure and the target distance were small^27^. The pressure at the nozzle part is low due to the contraction of the thin nozzle, while the velocity is high^28^. In the case of hydro-cutting technology, experimental results showed that the operating time and the distance of the water jet have significant effects on the effectiveness of coal face mining^29^. The frequency changes in an oscillating cavity are primarily caused by the variation of vortices. The development of vortex structures is consistent with the frequency changes^30^. By applying shock vibration and pulse jet, the stress condition of the rock bottom is improved, the cleaning of rock debris at the bottom hole is strengthened, and the rock breaking efficiency is increased^31^. When the impact frequency is close to the resonance frequency of the rock, the displacement response amplitude is the most significant^32^. It is necessary to comprehensively consider the influence of pulse frequency and pressure amplitude on the resonance effect to obtain a better rock-breaking effect^33^. It is concluded that high-frequency and low-amplitude harmonic, dynamic loads can improve the dynamic characteristics of the resonance impact drilling system, thereby improving the drilling efficiency^34^.

Numerous investigations have been carried out to examine the structure of the jet flow field and identify the key factors influencing rock-breaking efficiency, with a primary focus on direct waterjet impingement on the rock^35^. Moreover, several studies have been conducted to achieve impact rock-breaking using mechanical percussion tools, which often possess complex structures and generate lower impacting frequencies^36^. In this paper, combining the advantages of resonance rock breaking and self-excited oscillating, it is proposed to use a self-excited oscillation cavity to generate high-frequency impulse waterjet which can be equivalent to the natural frequency of hard rock (more than 200 Hz)^2,3,37^. Subsequently, the resonance between the impulse jet and the rock will be generated to achieve the resonance rock-breaking of hard rock. This approach significantly differs from rock-breaking by waterjet or mechanical percussion methods. This study proposes a pulse jet that uses self-excited oscillation to break rocks through resonance impact, which can significantly reduce the rock-breaking threshold strength and improve efficiency compared to high-pressure water jet and mechanical impact methods. The tool has minimal activities and sealing parts, resulting in a longer tool life. Previous studies have verified the effectiveness of jet impact resonance^15,16,33^. In this study, the flow field and impulse mechanism of the self-excited oscillating cavity were simulated using the large eddy simulation method of Computational Fluid Dynamics (CFD). An experimental bench for a full-scale self-excited oscillating tool was established to analyze the effects of length-diameter ratio, cavity thickness, and displacement on the cavity’s impulse characteristics through experiments and numerical simulations.

## Experimental

In the current work, a field-scale experimental apparatus was developed and used to carry out the impulse characteristics experiments induced by self-excited oscillation.

### Materials

**Figure 1:**
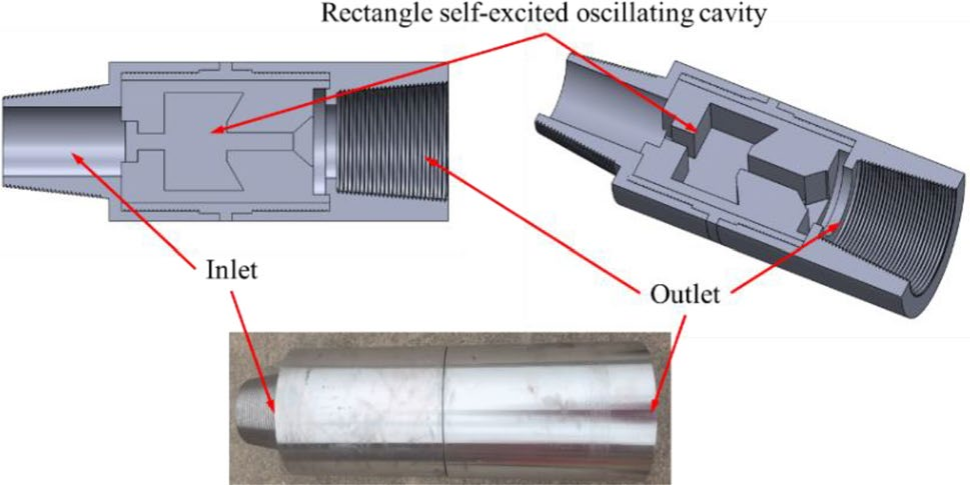
Structure and picture of self-excited oscillating tool.

The structure and picture of the self-excited oscillating tool are shown in Fig. 1. The self-excited oscillating tool primarily consists of three components: an inlet, a rectangular self-excited oscillating cavity, and an outlet. The inlet serves as the fluid inlet, while the rectangular self-excited oscillating cavity is used for generating the high-frequency pulse jet. Finally, the outlet functions as the pulse fluid outlet, and tap water is used as the fluid. During the experiments, tap water with a density of 1.0 g/cm^3^ was used as the liquid phase.

### Experimental apparatus

**Figure 2:**
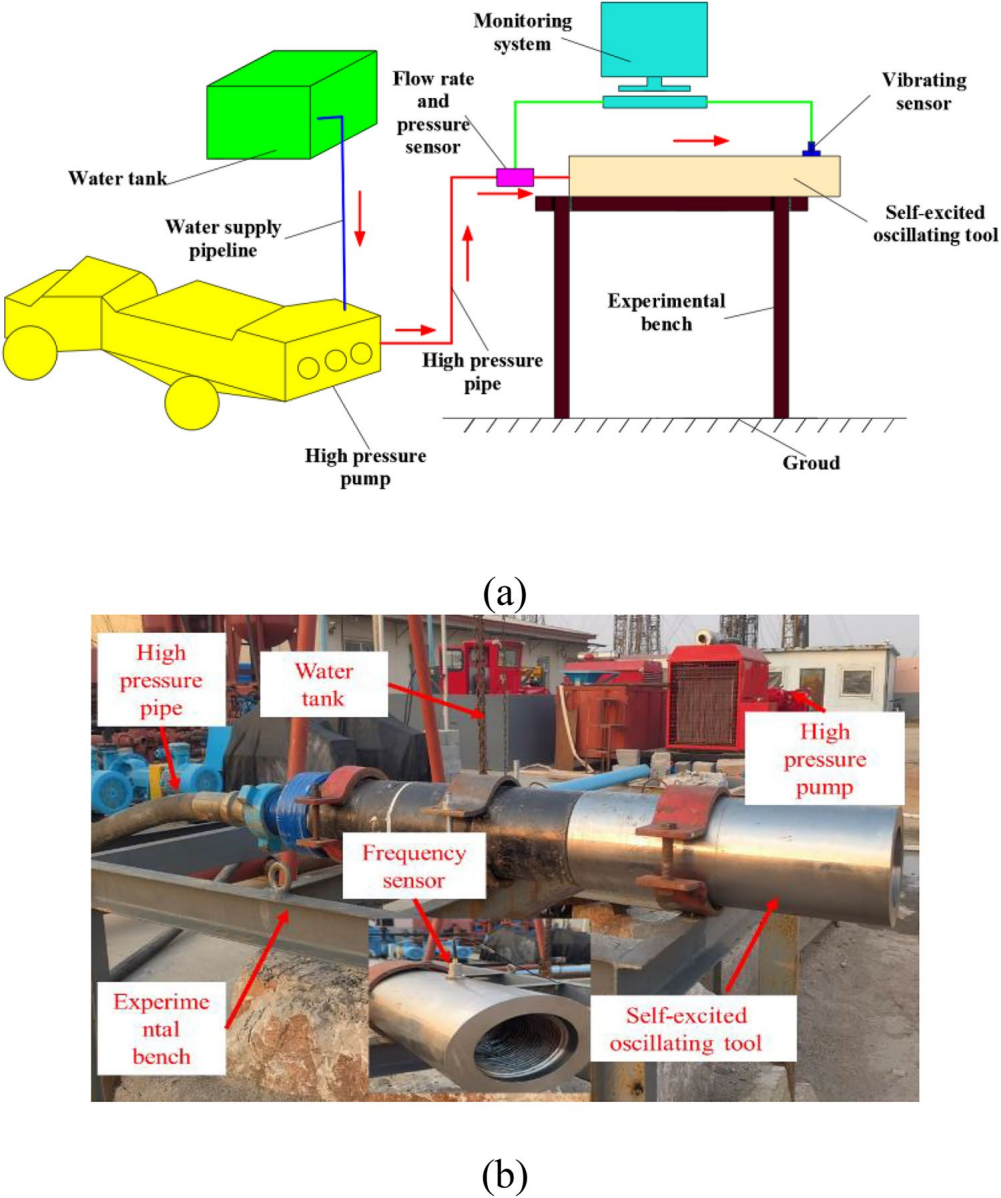
(**a**) Flow chart and (**b**) image of the experimental apparatus.

The experimental setup for the self-excited oscillating tool is shown in Fig. 2. The setup consists primarily of three systems: the fluid supply system, the experimental bench system, and the measuring system. The fluid supply system comprises a water tank, a fluid supply line, a high-pressure pump, and a high-pressure line. The water tank, with a volume of 15 m^3^, is the liquid storage container. The fluid supply line, with an inner diameter of 101.6 mm, is used for transporting the low-pressure fluid to the high-pressure pump. The high-pressure pump used is a plunger-type pump with a power of 320 kW, capable of producing a maximum pressure of 35 MPa. The high-pressure line, with a diameter of 50.8 mm, is used to transport the high-pressure water flow into the experimental bench system. The experimental bench system includes an experimental bench and a self-excited oscillating tool. The experimental bench, weighing 3.5 tons, is used to secure the self-excited oscillating tool and install the test sensor. The self-excited oscillating tool, with an outer diameter of 178 mm and a length of 0.73 m, is utilized to generate a high-frequency oscillating jet. The self-excited oscillating tool is fixed to the experimental bench by three screw hoops. The measuring system consists of a vibration sensor, a pressure sensor, a flow sensor, and a computer. The vibration sensor is capable of testing the pulse frequency of the self-excited oscillating tool, with a test frequency range of 1–5000 Hz and a sensitivity of 52.35 mV/g. The vibration sensor is a kind of piezoelectric acceleration sensor. The ceramic crystal induces the amount of charge, and the vibration frequency is obtained by subsequent integral processing. The uncertainty of conventional vibration sensors is mainly affected by the temperature and electromagnetic environment. Still, the piezoelectric acceleration sensor used in this experiment is not affected by external electromagnetic and temperature, and the measurement stability and accuracy are high. The pressure sensor, with a measuring range of 0–60 MPa and an accuracy of ± 0.1%, is used to measure the pressure of the self-excited oscillating tool. The uncertainty of the pressure sensor is mainly affected by the temperature and the conveying medium. The temperature of this experiment is the average temperature, and the conveying medium is clean water without impurities, so the measurement stability and accuracy can meet the experimental requirements. The flow sensors are used to measure the fluid flow rate of the self-excited oscillating tool, with a range of 0–150 m^3^/h and an accuracy of 0.5 level. The uncertainty of the flow sensor is mainly affected by the temperature, conveying medium, and vibration. The temperature of this experiment is normal temperature, and the conveying medium is clean water without impurities. The vibration of the flow sensor is small as it is fixed on the experimental bench with the screws. So, the measurement stability and accuracy of the flow sensor can meet the experimental requirements. The computer is used for receiving and processing the sensor signals. The flow direction of the fluid, described by the red arrows, is depicted in Fig. 2a. The tap water stored in the water tank is inhaled by the high-pressure pump through the water supply pipeline. Then, water is pressured by the high-pressure pump and transported to the flow rate and pressure sensor through the high-pressure pipe. Finally, the high-pressure water flow arrives and ejects from the self-excited oscillating tool.

## Numerical simulation

The study aims to investigate the impulse characteristics and mechanism generated by the self-excited oscillating cavity and to achieve a higher impulse frequency exceeding 200 Hz. In this paper, the flow field and impulse characteristics of the self-excited oscillating cavity were simulated using a large eddy simulation method of computational fluid dynamics. The analysis focused on evaluating the effects of length-diameter ratio, cavity thickness, and displacement on the impulse characteristics of the self-excited oscillating cavity.

### Governing equations

The large eddy simulation (LES) is a significant numerical simulation method in fluid dynamics that has been developed in recent decades. In LES, the flow field is filtered to remove vortices smaller than a certain scale, and only the large-scale vortices are calculated. The solution for this vortex is obtained by solving the additional equation. Typically, the grid-scale is used as the filter scale. The advantage of the large eddy simulation method is that it is more computationally efficient and requires fewer system resources compared to directly solving the Reynolds-averaged Navier–Stokes (RANS) equation or the Direct Numerical Simulation (DNS) equation. Additionally, LES provides greater accuracy compared to turbulence modeling methods^38^.

For this study, the continuity equation and the N–S equation are adopted as the basic governing equations for the large eddy simulation:

1 $$ \frac{\partial \rho }{{\partial t}} + \frac{\partial }{{\partial x_{i} }}(\rho \overline{u}_{i} ) = 0, $$

2 $$ \frac{\partial \rho }{{\partial t}}(\rho \overline{u}_{i} ) + \frac{\partial }{{\partial x_{j} }}(\rho \overline{u}_{i} \overline{u}_{j} ) = \frac{\partial }{{\partial x_{j} }}\left( {\mu \frac{{\partial \overline{u}_{i} }}{{\partial x_{j} }}} \right) - \frac{{\partial \overline{p}}}{{\partial x_{i} }} - \frac{{\partial \tau_{ij} }}{{\partial x_{j} }}. $$

In the three-dimensional rectangular coordinate system, the coordinate direction is denoted by *i* (*i* = 1, 2, 3). The velocity component direction in the three-dimensional rectangular coordinate system is denoted by *j* (*j* = 1, 2, 3) *u*_*i*_ and *u*_*j*_ are the velocity in the direction of *x*_*i*_ and *x*_*j*_ respectively; *t* is the time taken to flow through the cell; *ρ* is fluid density;* μ* is the dynamic viscosity of the fluid; *p* is the static pressure.

During the calculation, the N–S equation undergoes a filtering process. The filter function is used to decompose the flow variables into large scale quantities $\overline{f}(x,t)$ and small scale quantities $f^{\prime}(x,t)$:

3 $$ f(x,t) = \overline{f}(x,t) + f^{\prime}(x,t), $$

4 $$ \overline{f}(x,t){ = }\int {G(x,x^{\prime})} f(x^{\prime},t)dx^{\prime}, $$

where $G(x,x^{\prime})$ is the given kernel function, which is called filter function. Equation (3) is substituted into N–S equation and continuity equation:

5 $$ \left\{ \begin{array}{*{20}l}\frac{{\partial u_{i} }}{\partial t} + \frac{{\partial (u_{i} u_{j}^{{}} )}}{{\partial x_{j} }} = - \frac{1}{\rho }\frac{\partial p}{{\partial x_{i} }} + \mu \frac{{\partial^{2} u_{i} }}{{\partial x_{i} \partial x_{j} }} \hfill \\ \frac{{\partial u_{i} }}{{\partial x_{i} }} = 0 \hfill \\ \end{array} \right.. $$

The large eddy simulation equation satisfying large scale quantity $f^{\prime}(x,t)$ can be obtained:

6 $$ \left\{ \begin{array}{*{20}l} \frac{{\partial \overline{u}_{i} }}{\partial t} + \frac{{\partial (\overline{u}_{i} \overline{u}_{j}^{{}} )}}{{\partial x_{j} }} = - \frac{1}{\rho }\frac{\partial p}{{\partial x_{i} }} + \mu \frac{{\partial^{2} u_{i} }}{{\partial x_{i} \partial x_{j} }}{ - }\frac{{\partial \tau_{ij} }}{{\partial x_{j} }} \hfill \\ \frac{{\partial u_{i} }}{{\partial x_{i} }} = 0 \hfill \\ \end{array} \right., $$

7 $$ \tau_{ij} = \mathop {u_{i} u_{j} }\limits^{\_\_\_\_\_} - \overline{u}_{i} \overline{u}_{j} , $$

where $\tau_{ij}$ is turbulence subgrid stress.

If $\tau_{ij}$ is the subgrid tension, the model must be closed:

8 $$ \tau_{ij} { - }\frac{1}{3}\tau_{kk} \delta_{ij} = - 2\mu_{t} \overline{S}_{ij} , $$

$$ \overline{S}_{ij} \equiv \frac{1}{2}\left( {\frac{{\partial u_{i} }}{{\partial x_{i} }} + \frac{{\partial u_{i} }}{{\partial x_{j} }}} \right),\;\mu_{t} { = }\rho L_{s}^{2} \left| {\overline{S}} \right|,\;L_{s} = {\text{min(}}\kappa d,C_{s} V^{1/3} {),}\;C_{s} = 0.1,\;\left| {\overline{S} } \right| \equiv \sim \sqrt {2\overline{S}_{ij} \overline{S}_{ij} } C_{s} , $$

where $\mu_{t}$ is subgrid turbulent viscous force; $\overline{S}_{ij}$ is the rotation of subgrid turbulence tensor; $L_{s}$ is the mixing length of the grid; $\kappa$ is a constant; *d* is the distance to the nearest wall; *V* is the volume of the cell^39–41^.

### Geometric modeling and meshing

**Figure 3:**
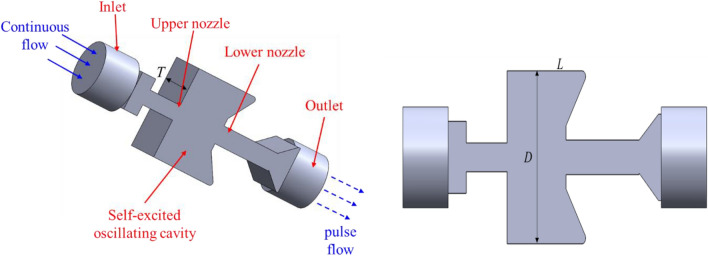
Physical model of impulse characteristics of the self-excited oscillating cavity.

To achieve the desired frequency modulation of the oscillating impulsion, the key structural parameters of the self-excited oscillating cavity underwent optimization. The physical model of impulse characteristics of a self-excited oscillating cavity is shown in Fig. 3. The continuous flow fluid enters the upper nozzle through the inlet, the jet from the upper nozzle enters the self-excited oscillating cavity, and the jet fluid continues to reach the lower nozzle. Due to the rebound and reflux effect of the wall at the lower nozzle, the continuous jet generated by the upper nozzle becomes a pulsed jet. Finally, the pulse flow is formed from the outlet after it is ejected from the lower nozzle. When the impact frequency of the pulsed jet is equivalent to the natural frequency of the rock, the rock begins to resonate, which can significantly reduce the rock-breaking threshold strength and improve the rock-breaking efficiency. Considering the 8-1/2″ well size, the space limitations in the downhole space, and the fluid up-flow, the outer diameter (*D*) of the oscillating cavity was set at 120 mm. The length-diameter ratio (*L/D*) varied within the range of 0.41 to 0.83. The cavity thickness (*T*) was adjusted between 20 and 70 mm, while the displacement ranged from 15 to 40 L/s. The cone angle of the downstream impact wall was set at 120°. Parameters for the impulse characteristics analysis of the self-excited oscillating cavity are depicted in Table 1. The SolidWorks 2018 software was used to construct the three-dimensional model.

**Table 1:** Parameters for the impulse characteristics analysis of self-excited oscillating cavity.

Parameters	Impulse characteristics analysis					
Length-diameter ratio, –	0.41	0.50	0.58	0.67	0.75	0.83
Cavity thickness, mm	20	30	40	50	60	70
Displacement, L/s	15	20	25	30	35	40

**Figure 4:**
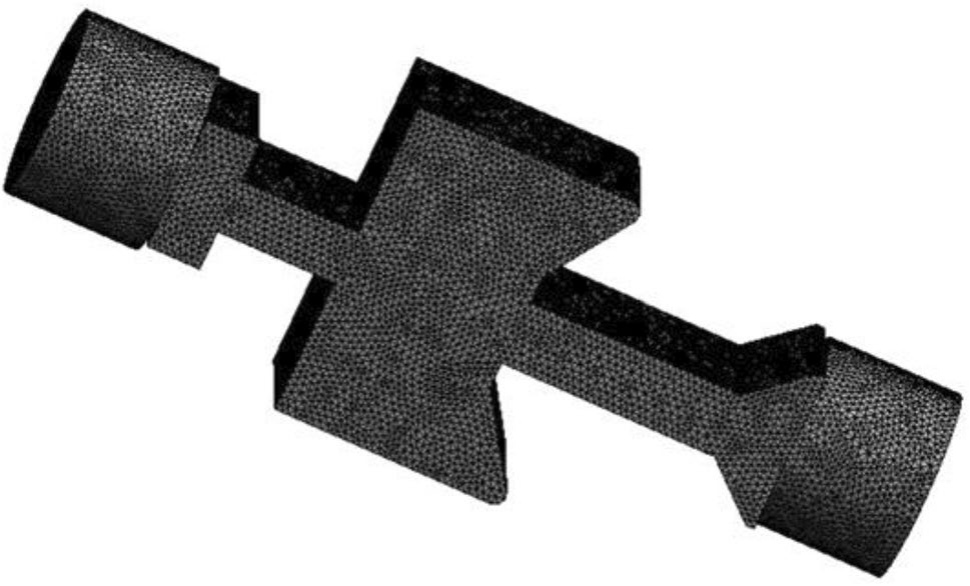
Meshing model of impulse characteristics of self-excited oscillating cavity.

The physical mode has meshed using the ANSYS ICEM software. Since large eddy simulation requires high-quality mesh, a hexahedral structured mesh was adopted to improve the overall mesh quality. The meshing model is shown in Fig. 4, and the total grid amount is 348,267.

### Model assumption and boundary condition

A model assumption was made, focusing solely on the high-frequency impulse characteristics and mechanism generated by the self-excited oscillating cavity, while disregarding the influence of temperature. The fluid flow simulation was carried out using ANSYS Fluent 18.0 software. The SIMPLEC technique was used to calculate the pressure field and velocity field, improving the convergence fitting. The control residual for each equation was maintained below 10^–5^ or the number of iterations was less than 5000. The pressure term was discretized using the standardized discrete method, while the convection and diffusion terms were discretized using the second-order upwind method. A turbulence intensity of 3% and a wall roughness of 10 μm were set^42^. The roughness constant was assigned the default value of 0.5, “velocity inlet” was designated as the inlet boundary, “reflect” was set for the wall boundary, and “pressure outlet” was specified for the outlet boundary^43^.

## Results and discussion

By simulating and analyzing the impulse modulation characteristics of the self-excited oscillating cavity with specific parameters (length–diameter ratio of 0.67, cavity thickness of 60 mm, and flow rate of 30 L/s), we can uncover the distribution patterns of the velocity field, pressure field, and turbulent kinetic energy field. This analysis enables us to clarify the impulse modulation mechanism of the self-excited oscillating cavity.

### Velocity field

**Figure 5:**
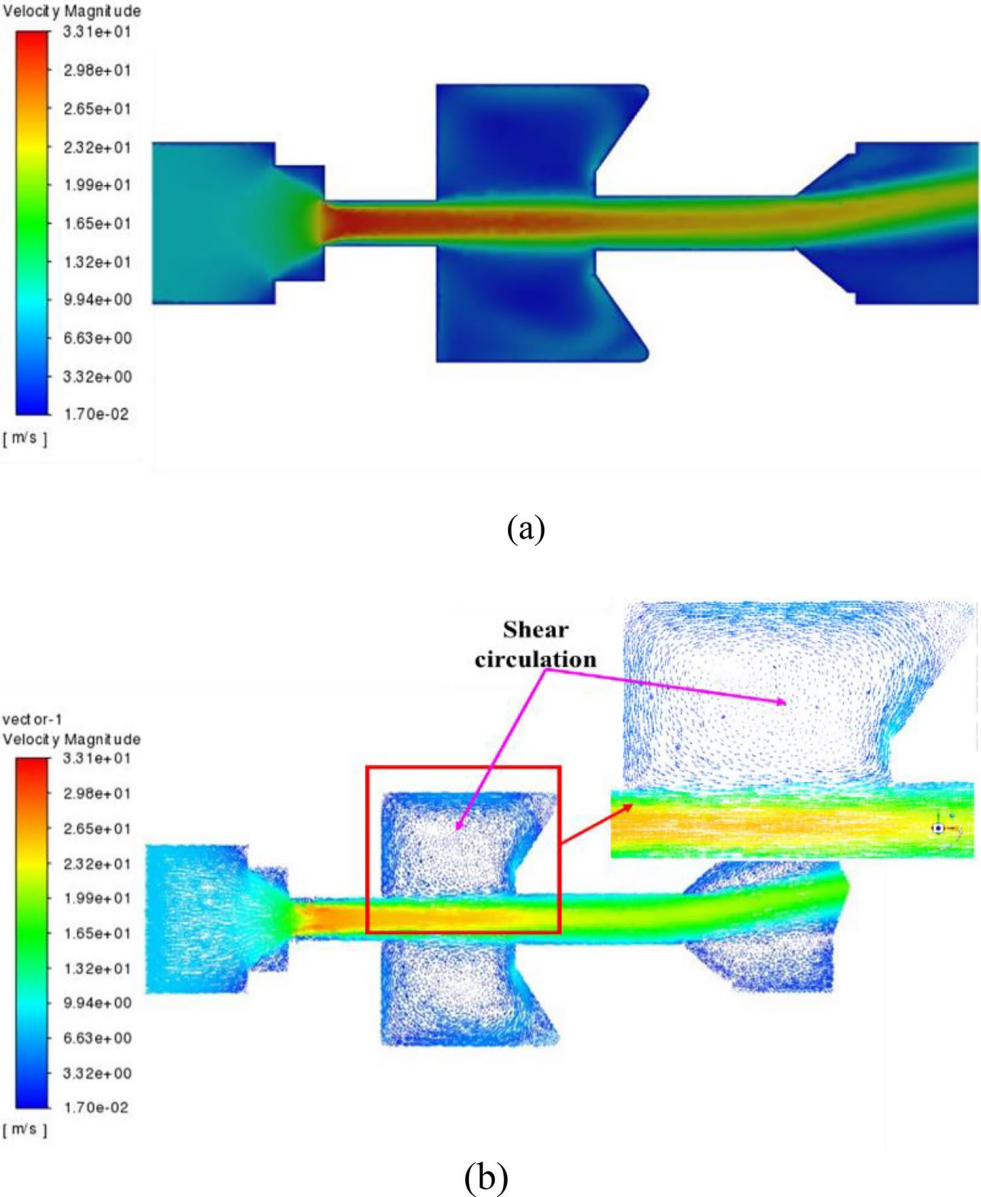
Velocity profile of self-excited oscillating cavity (**a**) velocity contour; (**b**) velocity vector.

The velocity profile of the self-excited oscillating cavity is shown in Fig. 5. The highest velocity is observed at the upper nozzle exit, reaching a value of 33.1 m/s as depicted in Fig. 5a. The interaction between the free shear jet at the upper nozzle outlet and the pressure disturbance wave generated by the collision feedback of the lower nozzle wall leads to the formation of shear circulation and fluid oscillation, as shown in Fig. 5b. At the outlet of the self-excited oscillating cavity, the fluid undergoes deflection due to the pulsating oscillation of the fluid.

### Pressure field

**Figure 6:**
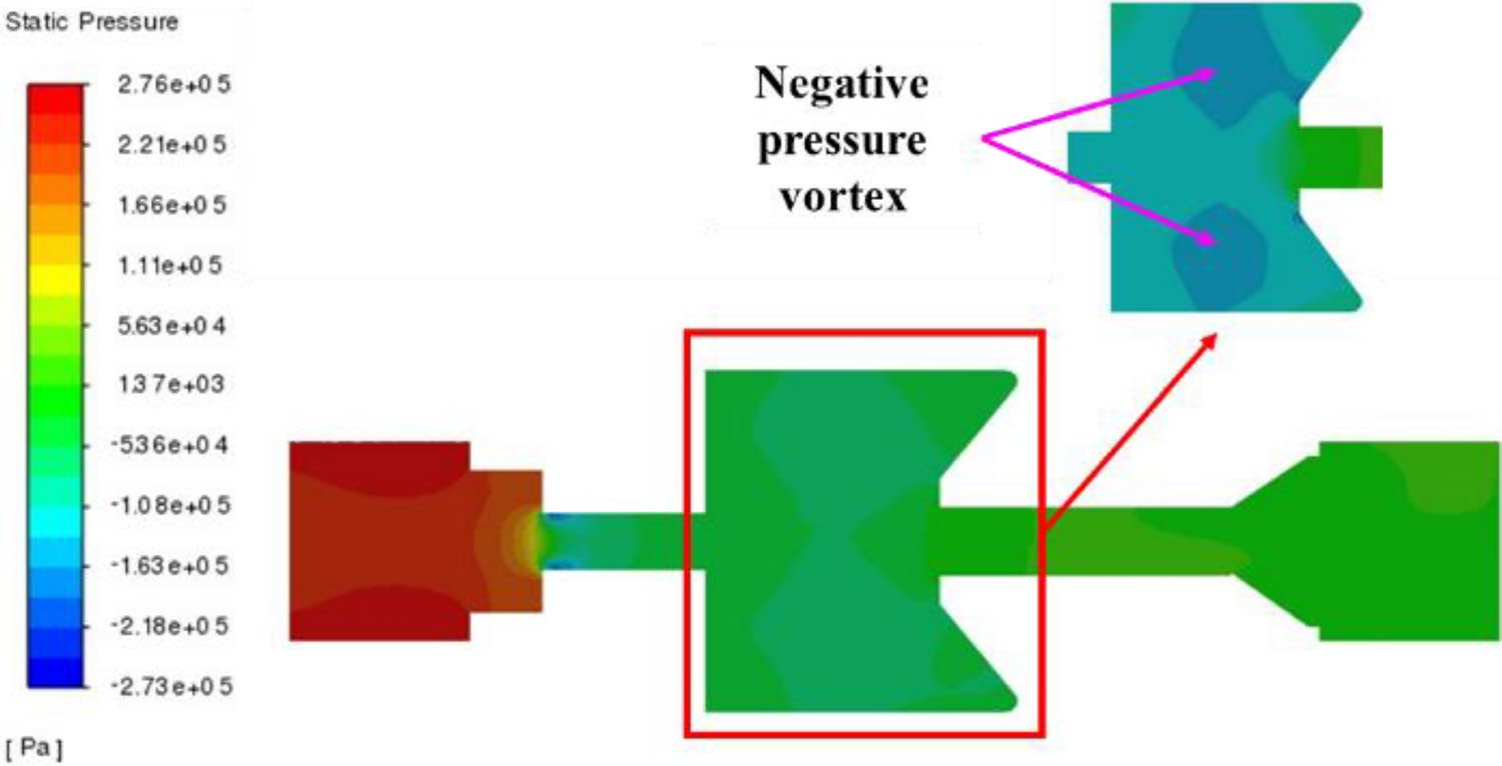
Pressure profile of self-excited oscillating cavity.

The pressure profile of the self-excited oscillating cavity is shown in Fig. 6. The maximum negative pressure value within the oscillating cavity is observed at the outlet of the upper nozzle, and there exists a region of negative pressure inside the oscillating cavity. As the fluid enters the oscillating cavity, it undergoes acceleration, resulting in a high-speed jet directed into the cavity. Due to the diverging flow of the jet within the cavity, when it reaches the lower section of the oscillating cavity, a portion of the fluid collides with the lower impact wall, subsequently following upward along the oscillating cavity wall. It then collides with the upper impact wall, generating a vortex ring around the central jet beam within the oscillating cavity. This vortex ring, moving at high speed, forms a central area of low pressure in the cavity’s center. The presence of these vortex rings exerts a damping effect on the fluid flowing through the middle region between the two vortex rings.

### Turbulent kinetic energy

**Figure 7:**
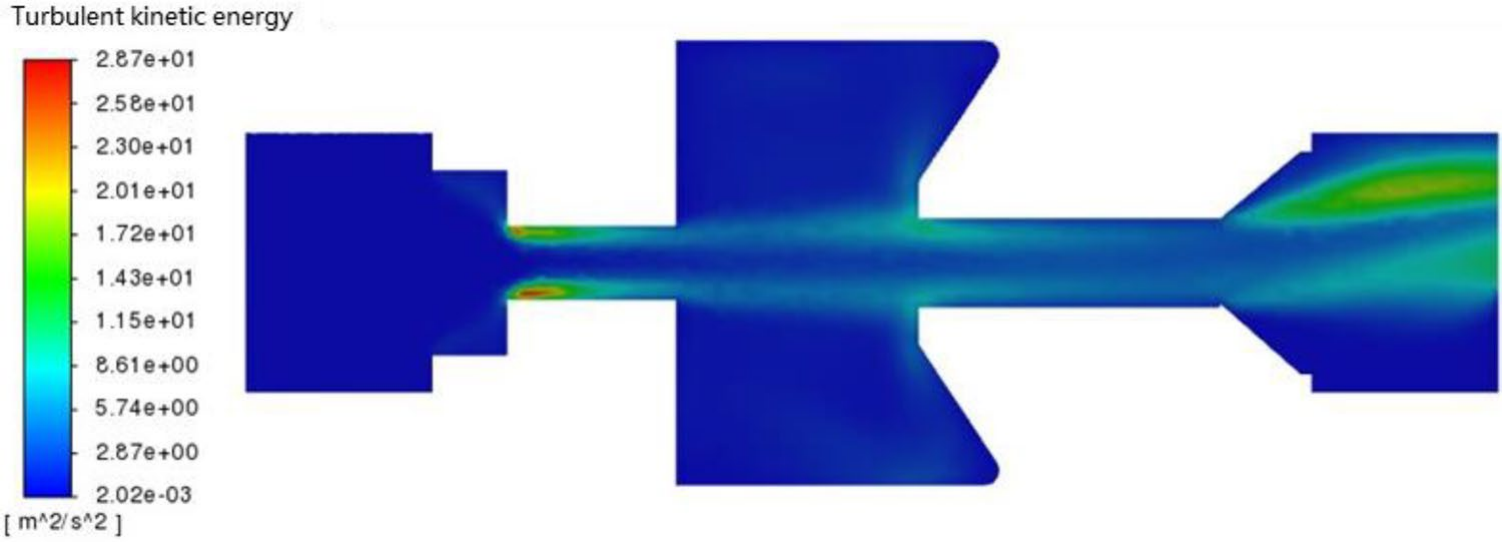
Turbulent kinetic energy profile of self-excited oscillating cavity.

The turbulent kinetic energy profile of the self-excited oscillating cavity is depicted in Fig. 7. The region with the highest turbulent kinetic energy is observed on both sides of the lower nozzle and at the outlet of the tool. This indicates that the fluid in these areas exhibits intense turbulence. Of particular note is the presence of a strong impulse jet at the exit. The flow velocity is higher and there is significant interaction between the fluids on both sides of the lower nozzle, leading to larger turbulent kinetic energy. As a result of the self-excited oscillation chamber, the fluid at the exit of the tool produces an obvious pulse effect. This makes the fluid pulsed fluid, hence the turbulent kinetic energy is large.

### Oscillation mechanism

**Figure 8:**
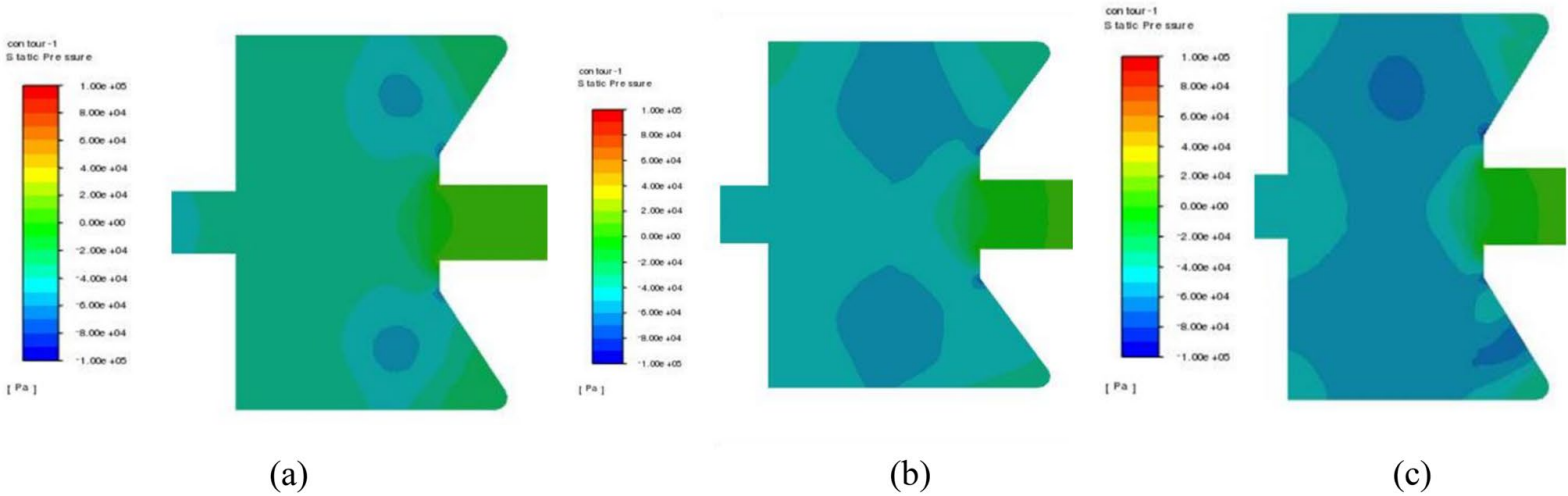
Time evolution of the pressure in self-excited oscillating cavity (**a**) 0.001 s; (**b**) 0.002 s; (**c**) 0.003 s.

The time evolution of the pressure in the self-excited oscillating cavity is shown in Fig. 8. A pair of symmetrical large-scale vortices is observed around the central jet area within the self-excited oscillating cavity, forming a low-pressure core area. By comparing the cloud images of the pressure field at different time intervals (0.001 to 0.003 s), it becomes apparent that the negative pressure vortex in the self-excited oscillating cavity initially forms near the impact wall of the cavity. It quickly develops into a symmetrical large-scale vortex. The vortex flows upstream along with the impact shear flow, moving periodically in a circumferential manner along the central axis and the cavity wall. The low-pressure vortices undergo a continuous cycle of occurrence, growth, collapse, and regeneration. Consequently, the size and shape of the vortices constantly change. These periodic changes in the fluid impedance within the oscillating cavity have an impact on the central main jet area. This, in turn, influences the pressure and velocity at the outlet of the lower nozzle, resulting in the formation of a self-excited oscillating impulse jet.

### Impulse frequency

**Figure 9:**
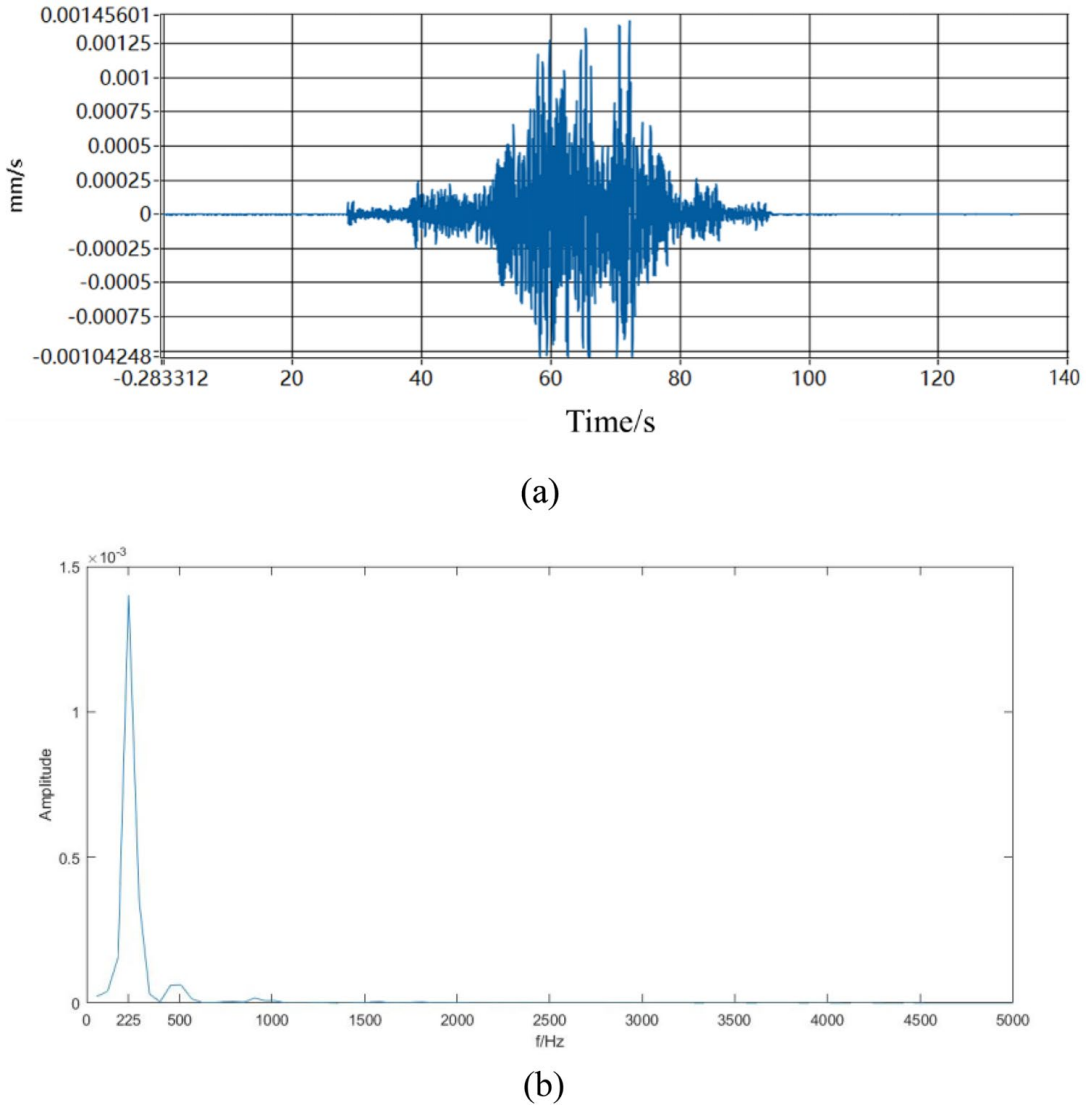
Impulse frequency generated by the self-excited oscillating tool (**a**) original frequency diagram and (**b**) frequency diagram after Fourier change.

The impulse frequency generated by the self-excited oscillating tool is shown in Fig. 9. In Fig. 9a, it is observed that before the high-pressure pump is activated, the velocity amplitude and frequency of the tool are zero. However, as the pump displacement increases, the velocity amplitude and frequency of the tool continuously rise. When the pump displacement reaches its maximum, the tool’s velocity amplitude and frequency also reach their maximum. As the pump displacement starts to decrease, the tool’s velocity amplitude and frequency gradually decrease as well. When the pump is turned off, the velocity amplitude and frequency of the tool gradually decrease until reaching zero. By performing a Fourier transform on the original vibration waveform shown in Fig. 9a, the impact frequency graph is obtained which is shown in Fig. 9b. Figure 9b shows two main frequencies: one at 225 Hz and the other around 500 Hz. Since the amplitude of 500 Hz is small, it can be assumed that the frequency generated by the self-excited oscillating tool can reach 225 Hz. This is based on the assumption that the length-diameter ratio is 0.67, the cavity thickness is 60 mm, and the flow rate is 25 L/s. This frequency exceeds the frequency requirement for resonance rock breaking, which is set at (200 Hz).

### Validation

Validation results for CFD simulation are shown in Table 2. The frequency generated by the self-excited oscillating tool experiments can reach 225 Hz (assuming a length-diameter ratio is 0.67, a cavity thickness of 60 mm, and a flow rate of 25 L/s). The frequency calculated by the simulation is 240 Hz (assuming a length-diameter ratio is 0.67, a cavity thickness of 60 mm, and a flow rate of 25 L/s). Previous studies have also shown that the self-excited oscillating cavity can generate high frequency pulse oscillating fluid^16,31^. The frequency obtained from the ground test is slightly lower than the result from the simulation, with a deviation of 6.67%, which falls within an acceptable range. The test results have confirmed the accuracy of the numerical model. This deviation could be attributed to the fluid pressure loss between the pressure gauge and the self-excited oscillating tool, resulting in a decreased frequency.

**Table 2:** Validation results for CFD simulation.

Method	Results (Hz)	Deviation	Conditions
Experiments	225	6.67%	Length–diameter ratio is 0.67, a cavity thickness of 60 mm, and a flow rate of 25 L/s
CFD simulation	240	–	Length–diameter ratio is 0.67, a cavity thickness of 60 mm, and a flow rate of 25 L/s

### Effect of the oscillating cavity parameters

The effects of length–diameter ratio, cavity thickness, and displacement on impulse frequency and pressure loss of the self-excited oscillating cavity were analyzed, leading to the identification of optimal parameters. Since the experimental study on the influence of length–diameter ratio and cavity thickness on the pulse effect of the tool requires constant change of the tool’s structural parameters and costly, time-consuming experiments, numerical simulation research was conducted solely for these parameters. Once the optimized parameters for length–diameter ratio and cavity thickness were obtained through numerical simulation, the effect of displacement on the tool’s pulse effect was studied through simulation and experiments, with the experimental results serving as a verification for the numerical simulation outcomes.

#### Length–diameter ratio

**Figure 10:**
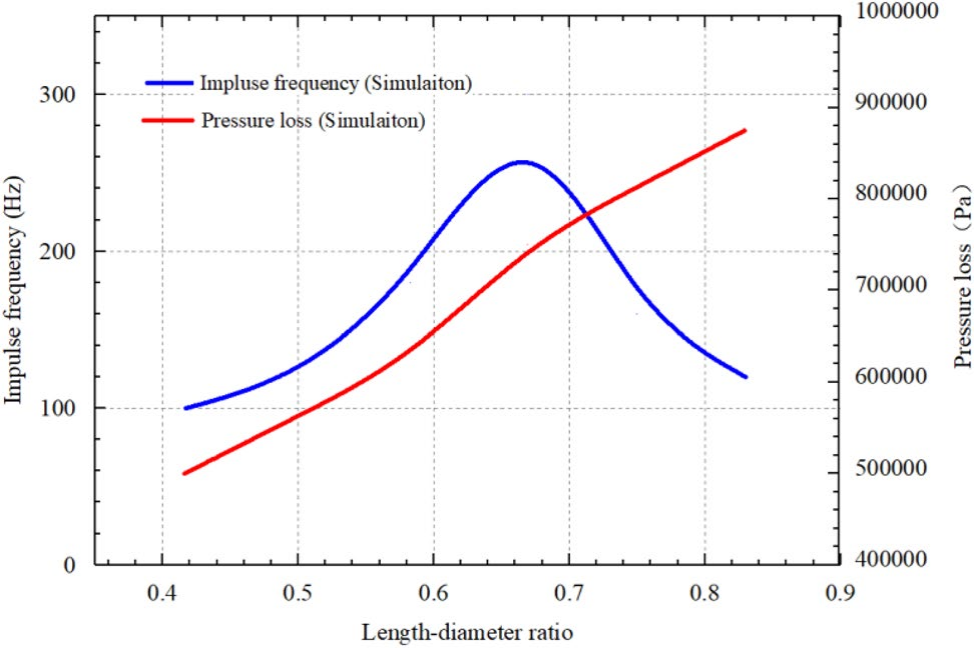
Effects of length-diameter ratio of the self-excited oscillating cavity on impulse frequency and pressure loss.

Figure 10 illustrates the effects of the length–diameter ratio of the self-excited oscillating cavity on impulse frequency and pressure loss, considering a cavity thickness of 60 mm and a flow rate of 30 L/s. The impulse frequency initially increases and then decreases with the increase in the length–diameter ratio of the oscillating cavity. The highest frequency, reaching 280 Hz, is achieved when the length–diameter ratio is 0.67. The cavity length directly influences the development of shear layer instability and perturbation feedback, which in turn affects the natural frequency of the oscillating cavity. If the cavity length is too short, it becomes challenging to establish a stable shear layer. Consequently, if the cavity length is too long, the feedback disturbance frequency components become excessive, resulting in ineffective excitation and increased energy dissipation in the jet. Additionally, as the length-diameter ratio increases, the pressure loss of the oscillating cavity also increases. This can be attributed to the larger movement distance of the fluid within the oscillating cavity associated with a higher length-diameter ratio, leading to increased pressure loss.

#### Cavity thickness

**Figure 11:**
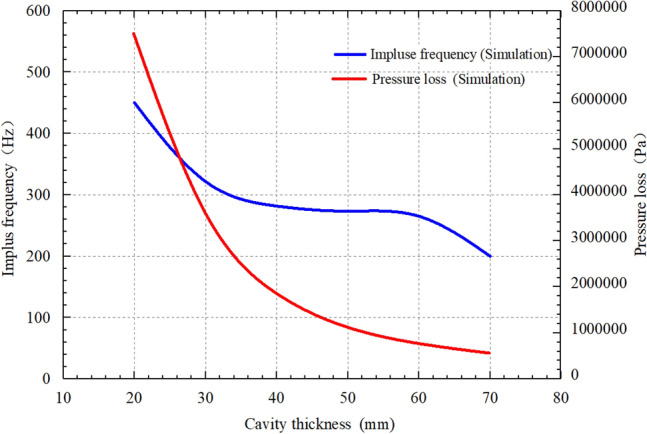
Effects of cavity thickness of the self-excited oscillating cavity on impulse frequency and pressure loss.

Figure 11 presents the effects of cavity thickness on impulse frequency and pressure loss of the self-excited oscillating cavity, considering a length-diameter ratio of 0.67 and a flow rate of 30 L/s. The impulse frequency of the oscillating cavity initially decreases, then remains unchanged, and subsequently decreases again as the cavity thickness increases. The cavity thickness exerts a significant influence on the impulse frequency of the self-excited oscillating cavity. When the cavity thickness increases (with a cavity diameter is less than 45 mm) the jet velocity within the oscillating cavity steadily decreases, resulting in reduced impact feedback from the jet and a subsequent decrease in the impulse frequency. The cavity thickness exerts a significant influence on the impulse frequency of the self-excited oscillating cavity. When the cavity thickness increases (with a cavity diameter of less than 45 mm), the jet velocity within the oscillating cavity steadily decreases, resulting in reduced impact feedback from the jet and a subsequent decrease in the impulse frequency. Once the cavity thickness exceeds 45 mm, the jet flow within the oscillating cavity stabilizes, leading to a constant impulse frequency. However, when the cavity thickness exceeds 60 mm, the vortex ring’s velocity with the oscillating cavity becomes too low, resulting in a decrease in the oscillation frequency and consequently diminishing the effect of the self-excited oscillation impulse jet. The pressure loss of the oscillating cavity decreases as the cavity thickness increases. With an increase in cavity thickness, the flow velocity of the jet within the oscillating cavity gradually decreases, resulting in a continuous reduction in pressure loss. Considering both the output frequency and pressure loss, the optimal cavity thickness is determined to be 60 mm.

#### Displacement

**Figure 12:**
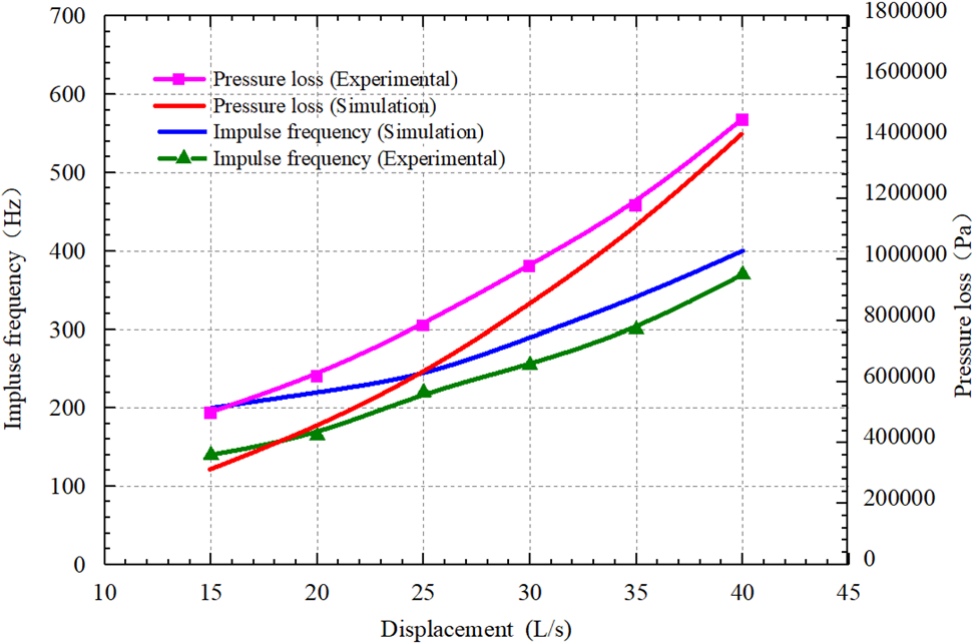
Effects of displacement of the self-excited oscillating cavity on impulse frequency and pressure loss.

Figure 12 illustrates the effects of displacement on impulse frequency and pressure loss of the self-excited oscillating cavity, considering a cavity thickness of 60 mm and a length-diameter ratio of 0.67. As the displacement increases, the impulse frequency also increases. This is due to the rising velocities of the inlet, interior, and outlet of the self-excited oscillating cavity, along with the shortened period of the negative pressure vortex within the cavity. The feedback time of the lower nozzle wall is shorter, while the feedback frequency of the jet at the upper nozzle is higher, resulting in an elevated pulse frequency. Consequently, as the displacement increases, the pressure loss also increases. This is attributed to the rapidly increasing fluid velocity within the self-excited oscillating chamber, continuous intensification of fluid motion turbulence, and subsequent augmentation of fluid energy consumption, leading to a continuous rise in tool pressure loss. Therefore, if field conditions permit, maximizing the displacement can enhance the impulse frequency.

## Conclusions

The flow field and impulse mechanism of the self-excited oscillating cavity were simulated using the large eddy simulation method. The influences of length-diameter ratio, cavity thickness, and displacement on the impulse characteristics of the self-excited oscillating cavity were analyzed, and the theoretical model was validated through experiments.

1. The upper nozzle outlet exhibits the maximum velocity and negative pressure within the cavity, while the maximum turbulence kinetic energy is observed on both sides of the lower nozzle and at the cavity outlet. At the outlet of the self-excited oscillating cavity, the fluid undergoes deflection due to the pulse oscillation. The high-speed vortex ring forms a central low-pressure area within the cavity, thereby damping the fluid flow through the middle of the two vortex rings. The size and shape of the low-pressure vortices constantly change, leading to periodic fluctuations in fluid impedance within the oscillating cavity.
2. Low-pressure vortexes undergo a cycle process of occurrence, growth, collapse, and regeneration, leading to continuous change in their size and shape. This cyclic behavior causes periodic fluctuations in fluid impedance within the oscillating cavity, which, in turn, affects the central main jet area and changes the pressure and velocity at the outlet of the lower nozzle.
3. Experimental validation of the theoretical model was conducted on the self-excited oscillating tool. The numerical simulation results were found to be in agreement with the experimental findings. The impulse frequency of the oscillating cavity initially increases and then decreases with the increase in the length-diameter ratio. The highest frequency is observed when the length-diameter ratio is 0.67. As the length diameter increases, the pressure loss of the oscillating cavity also increases. When it comes to the cavity thickness, the impulse frequency initially decreases, remains unchanged, and then decreases again as the thickness increases. Conversely, the pressure loss decreases with an increase in cavity thickness. A recommended cavity thickness of 60 mm is suggested. Additionally, increasing the displacement leads to an increase in both impulse frequency and pressure loss. As the pump displacement increases, the velocity amplitude and frequency of the oscillating cavity tool continue to rise.
4. The method used in this paper, known as large eddy simulation, is typically employed for numerical simulations of turbulent flow with high Reynolds numbers. The theoretical basis of this method is that there are inertial subscale vortices in high Reynolds number turbulent flow. Therefore, it is not suitable for numerical simulation of low Reynolds number flows. In practical engineering, the complexity of on-site construction conditions necessitates modulation of the structure and construction parameters of the tool to achieve resonance rock breaking and to obtain a better rock-breaking effect. In future studies, it is essential to continue optimizing the tool structure and improving the energy utilization efficiency.

## Data Availability

The data will be made available by the corresponding author upon reasonable request.

## List of symbols

*d*: Distance to the nearest wall (m)
*D*: Diameter of the oscillating cavity (m)
*L*: Length of the oscillating cavity (m)
*i*: Coordinate direction in the three-dimensional rectangular coordinate system (1, 2, 3)
*j*: Direction of the velocity component in the three-dimensional rectangular coordinate system (1, 2, 3)
$L_{s}$: Mixing length of the grid (m)
$P^{{}}$: Static pressure (Pa)
$\overline{S}_{ij}$: Rotation of subgrid turbulence tensor (–)
*t*: Time taken to flow through the cell (s)
*T*: Thickness of the oscillating cavity (m)
$u_{i}$: Velocity component in the *i* direction (m/s)
*u*_*j*_: Velocity component in the *j* direction (m/s)
*V*: Volume of the cell (m^3^)
*ρ*: Fluid density (kg/m^3^)
*μ*: Dynamic viscosity of the fluid (Pa s)
$\mu_{t}$: Subgrid turbulent viscous force (N)
$$\kappa$$: Constant (–)

## References

[1] Chang, D. Y. *et al.* The stress field of bottom hole in deep and ultra-deep wells. *Acta Pet. Sin.* **32**, 697–703 (2011).

[2] Zeng, Y. J. & Liu, J. L. Technical status of developmental trend of drilling techniques in deep and ultra deep well. *Pet. Drill. Tech.* **33**(5), 1–5 (2005).

[3] Wang, D. X. *et al.* Deep rock mechanics and deep or urltra deep well drilling technology. *Drill. Prod. Technol.* **29**, 6–10 (2005).

[4] Su, Y. N. *et al.* Status and research suggestions on the drilling and completion technologies for onshore deep and ultra deep wells in China. *Drill. Prod. Technol.* **42**, 527–542 (2020).

[5] Tibbitt, G. A. & Galloway, G. G. Particle drilling alters standard rock-cutting approach. *World Oil* **229**, 37–44 (2008).

[6] Zhao, J. *et al.* Cement erosion process with recyclable sand particle water-jet impacts. *J. Clean. Prod.* **292**, 126006 (2021). 10.1016/j.jclepro.2021.126006

[7] Ren, F. S., Fang, T. C. & Cheng, X. Z. Study on rock damage and failure depth under particle water-jet coupling impact. *Int. J. Impact Eng.* **139**, 103504 (2020). 10.1016/j.ijimpeng.2020.103504

[8] Wang, P., Li, Z. N., Ni, H. J., Liu, Y. D. & Dou, P. Experimental study of rock breakage of an interrupted pulsed waterjet. *Energy Rep.* **6**, 713–720 (2020). 10.1016/j.egyr.2020.03.018

[9] Li, G. S. & Shen, Z. H. Advances in researches and applications of water jet theory in petroleum engineering. *Pet. Explor. Dev.* **2**, 96–99 (2005).

[10] Hu, Z. J., Fan, Y. P. & Wang, J. Analysis on technical principle and development of resonance-enhanced drilling. *Oil Field Equip.* **47**, 72–78 (2018).

[11] Pavlovskaia, E., Hendry, D. C. & Wiercigroch, M. Modelling of high frequency vibro-impact drilling. *Int. J. Mech. Sci.* **91**, 110–119 (2015). 10.1016/j.ijmecsci.2013.08.009

[12] Ni, H. J., Wang, R. H. & Zhang, Y. Q. Numerical simulation study on rock breaking mechanism and process under high pressure water jet. *Appl. Math. Mech.* **26**, 595–1604 (2005). 10.1007/BF03246268

[13] Tang, C. L., Hu, D. & Pei, J. H. Experimental study on the frequency characteristic of the self-excited oscillation pulsed nozzle. *Acta Pet. Sin.* **28**, 122–125 (2007).

[14] Wang, X. C. *et al.* An experimental study on the jet pressure performance of organ–Helmholtz (O–H), self-excited oscillating nozzles. *Energies* **13**, 367 (2020). 10.3390/en13020367

[15] Deng, Y. J. *et al.* Study on the pulse frequency and pressure amplitude of self-oscillation pulsed supercritical carbon dioxide jet at different target distances. *Int. J. China Coal Soc.*10.13225/j.cnki.jccs.2023.0508 (2023). 10.13225/j.cnki.jccs.2023.0508

[16] Zhang, J. C., Zhang, B., Liu, B. & Li, B. Investigation on the influence of the frequency of pulsed water jet on the rock-breaking effect. *Powder Technol.* **431**, 119054 (2024). 10.1016/j.powtec.2023.119054

[17] Lin, B. Q., Zou, Q. L., Liang, Y. P., Xie, J. & Yang, H. M. Response characteristics of coal subjected to coupling static and waterjet impact loads. *Int. J. Rock Mech. Mining Sci.* **103**, 155–167 (2018). 10.1016/j.ijrmms.2018.01.032

[18] Li, J. B. *et al.* The self-propelled force model of a multi-orifice nozzle for radial jet drilling. *J. Nat. Gas Sci. Eng.* **24**, 441–448 (2015). 10.1016/j.jngse.2015.04.009

[19] Wang, H. Z. *et al.* Experiment on rock breaking with supercritical carbon dioxide jet. *J. Pet. Sci. Eng.* **127**, 305–310 (2015). 10.1016/j.petrol.2015.01.006

[20] Dehkhoda, S. & Hood, M. An experimental study of surface and sub-surface damage in pulsed water-jet breakage of rocks. *Int. J. Rock Mech. Min. Sci.* **63**, 138–147 (2013). 10.1016/j.ijrmms.2013.08.013

[21] Mao, X. J., Zhang, C. L., Wu, M. P., Ma, C. L. & Wang, Q. L. Application of a water jet for cleaning grease and improving the surface adhesion properties of galvanized steel wire ropes. *Sci. Rep.* **12**, 9680 (2022). 35690643 10.1038/s41598-022-13955-y PMC9188551

[22] Raghu Prasad, B. K. & Vidya Sagar, R. Relationship between AE energy and fracture energy of plain concrete beams: Experimental study. *J. Mater. Civil Eng.* **20**, 212–220 (2008). 10.1061/(ASCE)0899-1561(2008)20:3(212)

[23] Habak, M. & Lebrun, J. L. An experimental study of the effect of high-pressure water jet assisted turning (HPWJAT) on the surface integrity. *Int. J. Mach. Tools Manuf.* **51**, 661–669 (2011). 10.1016/j.ijmachtools.2011.05.001

[24] Zhao, J., Zhang, G. C., Xu, Y. J., Wang, R. H. & Zhou, W. D. Mechanism and effect of jet parameters on particle waterjet rock breaking. *Powder Technol.* **313**, 231–244 (2017). 10.1016/j.powtec.2017.03.026

[25] Zhang, J. S. *et al.* Optimization design and analysis of bionic friction reducing nozzle in oil shale high-pressure jet mining. *Appl. Sci.* **12**, 8159 (2022). 10.3390/app12168159

[26] Lv, Y. Y., Feng, Y. X., Li, X. H. & Xiang, W. Y. Experiments on breaking rock with high pressure cavitating water jets. *J. Chongqing Univ.* **29**, 88–91 (2006).

[27] Chen, L. H., Gao, D. R., Cheng, M. Z., Cai, Y. & Guo, L. W. Effect of special-shaped nozzle structure on water jet performance. *Processes* **10**, 2066 (2022). 10.3390/pr10102066

[28] Dubinski, J. *et al.* In-situ experimental study on hydro-borehole technology application to improve the hard coal excavating techniques in coal mine. *Sci. Rep.* **13**, 1190 (2023). 36681710 10.1038/s41598-023-28501-7 PMC9867735

[29] Takagaki, N. *et al.* Estimation of high-speed liquid-jet velocity using a pyro jet injector. *Sci. Rep.* **9**, 19859 (2019). 31882780 10.1038/s41598-019-56511-x PMC6934512

[30] Liu, Y. *et al.* Study on the development law of self-oscillating pulsed SC-CO2 jet vortex structure and its effect on frequency. *Geomech. Geophys. Geo-Energy Geo-Resour.* **9**, 94 (2023). 10.1007/s40948-023-00641-0

[31] Lei, P. *et al.* Performance analysis and optimization for hydraulic components of self-oscillating rotary impact drilling tool. *J. Vib. Shock* **33**, 175–180 (2014).

[32] Song, H. Y. *et al.* Analysis and numerical simulation for resonant response of bottom hole rock. *J. Vib. Shock* **38**, 13–20 (2019).

[33] Liu, Y. *et al.* Study on the optimal target distance of self-oscillation pulsed SC-CO2 jet based on resonant effect. *Geoenergy Sci. Eng.* **229**, 212018 (2023). 10.1016/j.geoen.2023.212018

[34] Li, S. Q. *et al.* Dynamics of resonance impact drilling based on constant depth-of-cut and state-dependent time delay models. *J. China Univ. Pet. Ed. Nat. Sci.* **48**, 124–132 (2024).

[35] Momber, A. W. Effects of target material properties on solid particle erosion of geomaterials at different impingement velocities. *Wear* **319**, 69–83 (2015). 10.1016/j.wear.2014.07.007

[36] Wang, W. *et al.* Analysis and testing of the working characteristics of a thepulsating torsional impact drilling tool. *Pet. Drill. Tech.* **50**, 63–69 (2022).

[37] Li, S. Q. *et al.* Rock fragmentation mechanism and drilling performance of harmonic vibration-impact drilling technique. *J. China Univ. Pet. Ed. Nat. Sci.* **45**, 67–73 (2021).

[38] Piomelli, U. Large-eddy simulation: Achievements andchallenges. *Prog. Aerospace Sci.* **35**, 335–362 (1999). 10.1016/S0376-0421(98)00014-1

[39] Moser, R. D. Direct numerical simulation of turbulent channel flow up to R_eτ_=590. *Phys. Fluids* **11**(4), 943–945 (1999). 10.1063/1.869966

[40] Lilly, D. K. A proposed modification of the germano sub grid scale closure method. *Phys. Fluids A* **4**, 633–635 (1992). 10.1063/1.858280

[41] Zhou, B., Cui, G. X. & Chen, N. X. Dynamic procedure based on the scale-similarity hypotheses for largeeddy simulations. *J. Tsinghua Univ. Sci. Technol.* **46**(8), 1438–1441 (2006).

[42] Peng, W. S. & Cao, X. W. Rock Numerical simulation of solid particle erosion in pipe bends for liquid–solid flow. *Powder Technol.* **294**, 266–279 (2016). 10.1016/j.powtec.2016.02.030

[43] Zhao, J. *et al.* Experimental and theoretical evaluation of solid particle erosion in an internal flow passage within a drilling bit. *J. Pet. Sci. Eng.* **160**, 582–596 (2018). 10.1016/j.petrol.2017.10.068

